# Sterilizing activity of spectinamide MBX-4888A when replacing linezolid in the Nix-TB regimen in the relapsing BALB/c mouse model of tuberculosis

**DOI:** 10.1128/aac.01183-25

**Published:** 2025-09-30

**Authors:** Nathan Peroutka-Bigus, Michael S. Scherman, Firat Kaya, Samanthi L. Waidyarachchi, Jiuyu Liu, Joel N. Rushefsky, Michelle M. Butler, Terry Bowlin, Bernd Meibohm, Mercedes Gonzalez-Juarrero, Anne J. Lenaerts, Matthew Zimmerman, Richard E. Lee, Gregory T. Robertson

**Affiliations:** 1 Mycobacteria Research Laboratories, Department of Microbiology, Immunology and Pathology, Colorado State University 3447, Fort Collins, Colorado, USA; 2 Center for Discovery and Innovation, Hackensack Meridian School of Medicine 576909 , Nutley, New Jersey, USA; 3 Microbiotix, Inc. 375141 , Worcester, Massachusetts, USA; 4 Department of Chemical Biology & Therapeutics, St. Jude Children's Research Hospital 5417, Memphis, Tennessee, USA; 5 Department of Pharmaceutical Sciences, University of Tennessee Health Science Center 12326, Memphis, Tennessee, USA; City St George's, University of London, London, United Kingdom

**Keywords:** tuberculosis, relapse, BALB/c, spectinamide, bedaquiline, pretomanid

## Abstract

Spectinamides have garnered interest as experimental tuberculosis therapeutics owing to their safety profile and efficacy as partner agents when used in conjunction with established regimens in mice. The Nix-TB regimen of bedaquiline, pretomanid, and linezolid represents a short, effective regimen recommended for treatment of pre-extensively drug-resistant tuberculosis. However, linezolid administration is associated with severe adverse events that limit its use. Here we present preclinical data comparing Nix-TB regimens anchored by either linezolid or spectinamide MBX-4888A.

## INTRODUCTION

The effective treatment of patients with all forms of tuberculosis (TB) is crucial in the fight to eliminate the spread of drug-resistant *Mycobacterium tuberculosis* (*Mtb*). Combination antibiotic regimens intended for the treatment of patients with drug-resistant TB are associated with severe adverse events (AEs), which complicate treatment, leading to treatment discontinuation, non-compliance, and further expansion of drug resistance (1). The Nix-TB Phase 3 clinical trial demonstrated exceptional cure rates in patients with drug-resistant forms of TB using a three-drug regimen consisting of bedaquiline (B), pretomanid (Pa), and linezolid (L) (2). While the BPaL regimen is efficacious in treating drug-resistant forms of TB, long-term administration of L was associated with AEs of peripheral neuropathy, myelosuppression, optic neuritis, and anemia, leading to dose reductions and frequent interruptions in L treatment (2). Spectinamide MBX-4888A (4888A) represents a new class of semisynthetic antibiotics that shows promising activity against *Mtb* in diverse preclinical murine models of TB disease with favorable safety profiles (3–9). Like L, 4888A targets protein synthesis, but occupies a different binding site in the ribosome (5).

Spectinamides are promising partner agents when administered with standard combination TB treatment regimens. The inclusion of spectinamide Lee-1599 with BPa resulted in increased bactericidal activity in a high dose aerosol subacute TB BALB/c infection model (4); while 4888A, given by injection with the front line standard of care regimen, led to improved bactericidal responses and treatment shortening in the subacute relapsing BALB/c model and in the more rigorous chronic C3HeB/FeJ relapsing spectrum of disease model featuring heterogeneous advanced pulmonary disease (8). An inhaled formulation of spectinamide Lee-1599 showed similar efficacy, but with reduced toxicities and an absence of known hematological effects, when used as a replacement for L in the BPaL regimen in BALB/c and C3HeB/FeJ mice (9). However, those studies failed to address whether a spectinamide, when administered in place of L in BPaL, resulted in a durable sterilizing cure as measured by the prevention of relapse after treatment completion. This study employed the high-dose aerosol BALB/c relapsing mouse model to compare bactericidal responses and time to achieve durable cure for BPaL or BPa anchored by injectable compound 4888A.

**Fig 1:**
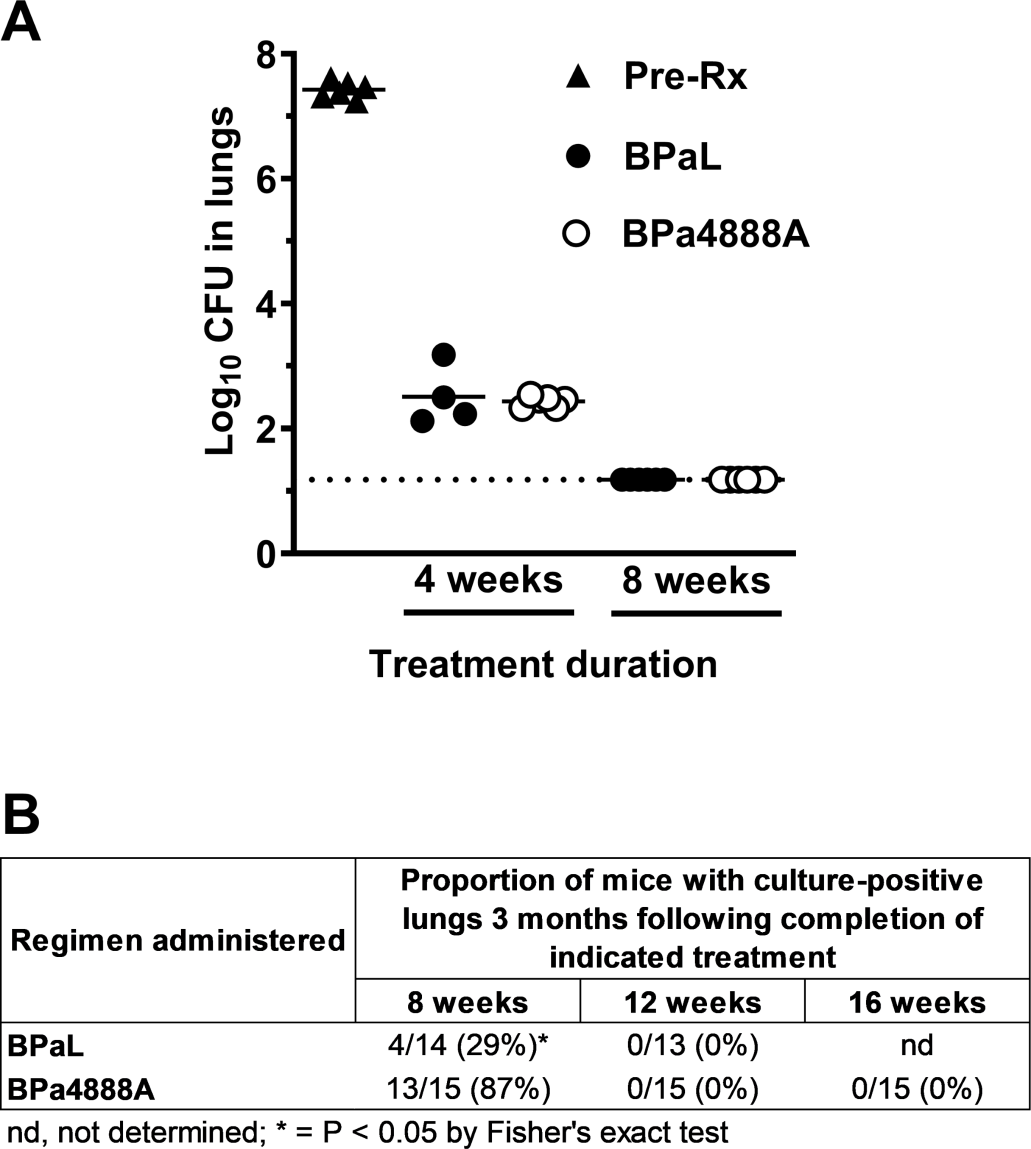
Antimicrobial regimen activity against *Mycobacterium tuberculosis* Erdman strain in the BALB/c relapsing mouse model following high-dose aerosol infection. (**A**) Bactericidal activity of the two regimens over the 8-week treatment course. CFU lung burdens at the start of treatment (closed triangles), or after 4 or 8 weeks of BPaL (closed circles), or 4 or 8 weeks of BPa4888A (open circles). (**B**) The number of mice that relapsed 3 months after the indicated treatment duration over the group total. The percentage of mice relapsing is listed in parentheses. Drug doses in mg/kg: (**B**) bedaquiline (25), (Pa) pretomanid (100), (L) linezolid (100), and (4888A) MBX-4888A (200).

One day following high dose aerosol with *Mtb* Erdman (see Methods in supplemental materials), average CFU lung burdens in female BALB/c mice were 4.04 log10 CFU and increased to 7.42 log10 CFU 10 days later at the start of treatment. During treatment, both BPaL and BPa4888A significantly reduced *Mtb* lung burdens. With 4 weeks of therapy (5 out of 7 days per week), BPa4888A reduced *Mtb* lung burdens by 4.98 log10 CFU, which was not significantly different from the 4.91 log10 CFU reduction with BPaL therapy (Fig. 1A; Table S1). Following 8 weeks of therapy, both treatments reduced *Mtb* lung burdens to below the limit of detection (<1.18 log10 CFU), suggesting the presumptive clearance of *Mtb* from the lungs (Fig. 1A; Table S1). Weight loss is associated with the severity of disease in this model. Following an initial period of weight decline, mice in both treatment arms gained weight throughout the remainder of the study (Fig. S1), indicating clinical improvements with treatment. Fewer relapse events were observed in mice held for 3 months after receiving 8 weeks of BPaL compared to those receiving 8 weeks of BPa4888A (29% vs 87% [*P* < 0.05]), with all mice achieving durable cure with 12 weeks of treatment (BPaL and BPa4888A) (Fig. 1B). The current standard-of-care (SOC) regimen, composed of 2 months of isoniazid (H), rifampin (R), pyrazinamide (Z), and ethambutol (E) in the intensive phase (2HRZE) and only HR in the continuation phase, was included in this study and published elsewhere (8); in comparison, the proportion of mice relapsing 3 months following 12 weeks of 2HRZE/HR treatment was 100% (15 out of 15 mice relapsed) (8) indicating that both test regimens, BPaL and BPa4888A, are superior to the SOC regimen in this model. Pharmacokinetic analysis based on sparse plasma sampling in infected (not shown) and healthy mice (Fig. S2) confirmed no drug-drug interactions with equivalent drug exposures for BPa and 4888A in plasma (Tables S2a through d).

Although effective, clinical toxicities of L necessitate dose reduction or dosing holidays (2), which poses a serious threat to further expansion of clinical resistance to B (10). Modifications to BPaL, as the current WHO-recommended pre-XDR therapy (11), are desirable to improve clinical control of TB. Although restricted currently to injection or inhaled therapies, spectinamides exhibit favorable safety profiles and tolerability in preclinical studies (3, 8, 9), and as shown here, appear capable of contributing to durable cure in the BALB/c relapsing mouse model as a companion drug to BPa. Indeed, in a previous study, 4888A was administered subcutaneously for 20 weeks in *Mtb*-infected mice, providing an additional data point regarding the safety and tolerability of spectinamide 4888A (8). While these results are presently limited to outcomes in the conventional BALB/c relapsing mouse model—thought to best represent uncomplicated TB disease—our prior work demonstrated effective distribution and accumulation of 4888A within the caseous lesions of C3HeB/FeJ mice (8), suggesting a possible role for BPa4888A in harder-to-treat patients with necrotic acellular caseum-filled granulomas; this remains to be tested.

While both BPaL and BPa4888A achieved a 100% cure rate after 12 weeks of treatment, BPaL proved superior to BPa4888A in preventing relapse after 8 weeks of therapy followed by 3 months of drug-free recovery, indicating more rapid clearance of residual drug-tolerant bacilli by the Nix-TB regimen. As both combination regimens were anchored by BPa, one possible explanation is the nearly 100% oral bioavailability of L in humans and mice (12, 13) improves early target attainment and drives cure in drug-tolerant populations that arise during drug treatment, but other possibilities such as absolute target potency and the differences in L (14) and 4888A (15) model-based exposure responses should be considered.

This study has several limitations. Despite similar bactericidal responses and the ability to achieve a durable cure by 12 weeks of treatment, evidence supporting the contribution of 4888A to the efficacy of BPa can only be inferred as the study lacked a direct BPa comparator for this purpose. However, previous work showed that a closely related spectinamide, Lee-1599, does add appreciably to the BPa backbone in this same infection model (4). As such, further studies are needed to de-convolute the contribution of 4888A to cure in this preclinical model. Additionally, while sparse PK assessment provided data that co-administration of 4888A with BPa did not result in any drug-drug interactions, a full PK analysis was not performed, and the comparative behaviors of L and 4888A in this context were not evaluated.

This study provides further evidence supporting the safety and efficacy of spectinamide 4888A. Given its unique binding site and absence of current clinical use, it is anticipated that 4888A will not be impacted by any pre-existing clinical resistance—which is not the case for linezolid (a repurposed drug), for which clinical resistance is on the rise (16, 17).

## SUPPLEMENTAL MATERIAL

Supplemental material aac.01183-25-s0001.docx Tables S1 and S2; Fig. S1 and S2; Supplemental methods.

ASM does not own the copyrights to Supplemental Material that may be linked to, or accessed through, an article. The authors have granted ASM a non-exclusive, world-wide license to publish the Supplemental Material files. Please contact the corresponding author directly for reuse.
