# Supplementary material for: Sterilizing activity of spectinamide MBX-4888A when replacing linezolid in the Nix-TB regimen in the relapsing BALB/c mouse model of tuberculosis
Source: Antimicrob Agents Chemother. 2025 Sep 30;69(11):e01183-25. doi: 10.1128/aac.01183-25 (PMC12587623; doi:10.1128/aac.01183-25)
Supplement: Supplemental material — Tables S1 and S2; Fig. S1 and S2; Supplemental methods. [file aac.01183-25-s0001.docx]

**Supplemental Data**

**Title: Sterilizing activity of spectinamide MBX-4888A when replacing linezolid in the Nix-TB regimen in the relapsing BALB/c mouse model of tuberculosis**

**Authors:**

Nathan Peroutka-Bigus^1^, Michael S. Scherman^1^, Firat Kaya^2^, Samanthi L. Waidyarachchi^3^, Jiuyu Liu^4^, Joel N. Rushefsky^1^, Michelle M. Butler^3^, Terry Bowlin^3^, Bernd Meibohm^5^, Mercedes Gonzalez-Juarrero ^1^, Anne J. Lenaerts^1^, Matthew Zimmerman^2^, Richard E. Lee^4^, and Gregory T. Robertson^1^

**Affiliation:**

^1^Mycobacteria Research Laboratories, Department of Microbiology, Immunology and Pathology, Colorado State University, Fort Collins, Colorado, USA.

^2^Center for Discovery and Innovation, Hackensack Meridian School of Medicine, Nutley, New Jersey, USA.

^3^Microbiotix, Inc., Worcester, Massachusetts, USA

^4^Department of Chemical Biology & Therapeutics, St. Jude Children’s Research Hospital, Memphis, Tennessee, USA

^5^Department of Pharmaceutical Sciences, University of Tennessee Health Science Center, Memphis, Tennessee, USA.

Keywords: Tuberculosis, relapse, BALB/c, spectinamide, bedaquiline, pretomanid

Running Title: Testing a non-oxazolidinone containing Nix-TB regimen

| **Table S1. Regimen efficacy at four- and eight-weeks treatment in BALB/c mice infected with *Mycobacterium tuberculosis* Erdman shown as the CFU lung burden.** | | | | | | | | | | |
| --- | --- | --- | --- | --- | --- | --- | --- | --- | --- | --- |
| **Regimen** | **weeks of treatment** | **Mouse log_10_ CFU lung burden** | | | | | | **n*** | **AVG** | **SEM** |
|  |  | **A** | **B** | **C** | **D** | **E** | **F** |  |  |  |
| **Day 1 post-infection** | **NA** | 4.07 | 4.18 | 4.07 | 3.99 | 4.08 | - | 10/10 | 4.04 | 0.03 |
|  |  | 4.01 | 4.11 | 3.94 | 3.94 | 3.93 | - |  |  |  |
| **Pretreatment** | **NA** | 7.61 | 7.47 | 7.53 | 7.24 | 7.32 | 7.38 | 6/6 | 7.42 | 0.06 |
| **BPaL** | **4** | 2.50 | c | 2.23 | c | 3.18 | 2.12 | 4/4 | 2.51 | 0.24 |
|  | **8** | <1.18 | <1.18 | <1.18 | <1.18 | <1.18 | <1.18 | 0/6 | <1.18 | 0.00 |
| **BPa4888A** | **4** | 2.46 | 2.33 | 2.46 | 2.49 | 2.33 | 2.54 | 6/6 | 2.44 | 0.04 |
|  | **8** | <1.18 | <1.18 | <1.18 | <1.18 | <1.18 | <1.18 | 0/6 | <1.18 | 0.00 |
| *** = number of mice with CFU/number of mice at the time of sacrifice.** | | | | | | | | | | |
| **c indicates plate contamination observed. CFU limit of detection is 1.18 Log_10_ bacteria.** | | | | | | | | | | |


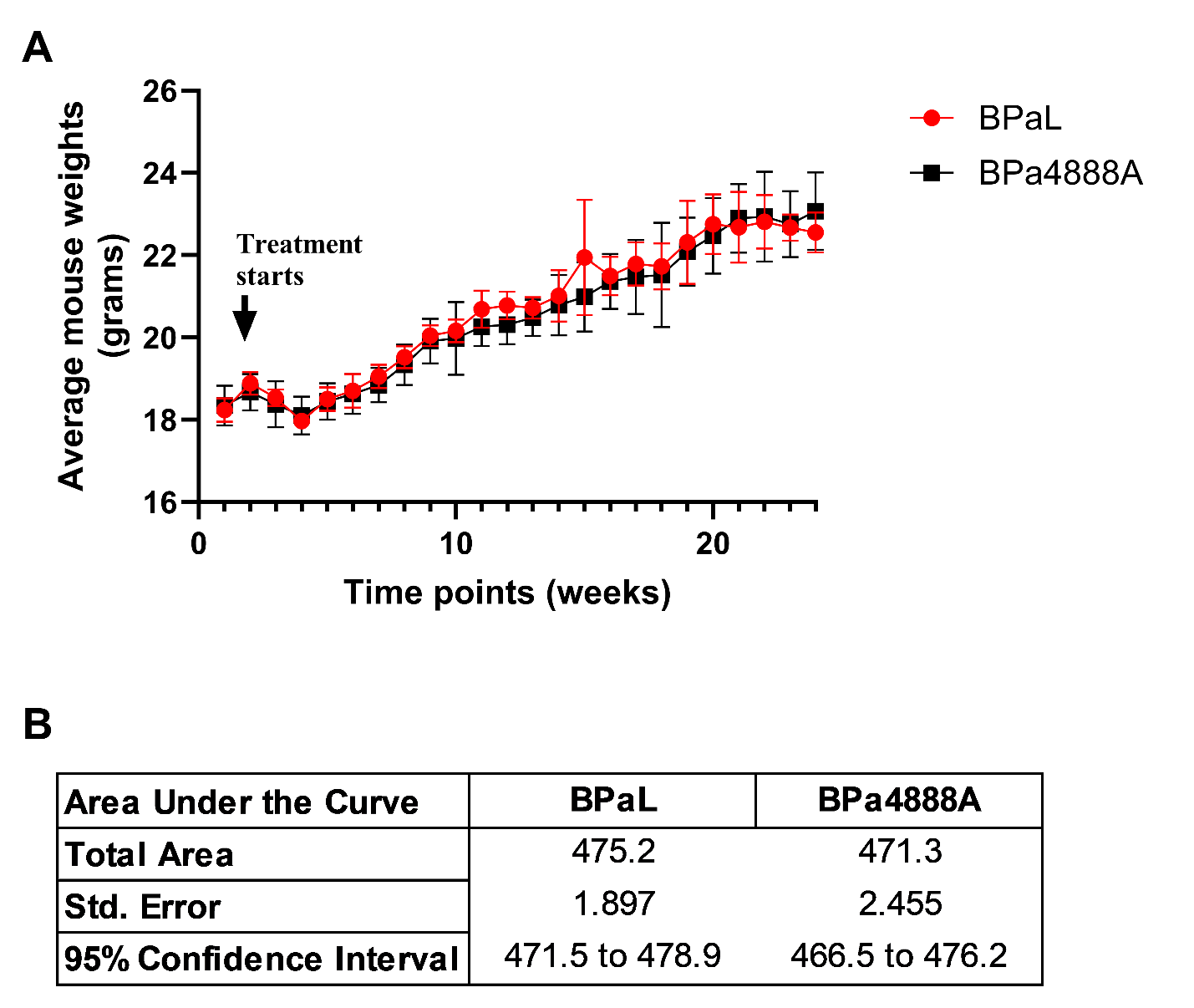


**Figure S1**. BALB/c mouse weights following high dose aerosol infection with *Mycobacterium tuberculosis* Erdman strain and subsequent treatment with either BPaL or BPa4888A. **A**. Average mouse weights over the experimental time course. **B**. Area Under the Curve analysis of the average mouse weights. GraphPad Prism 10.4.1.


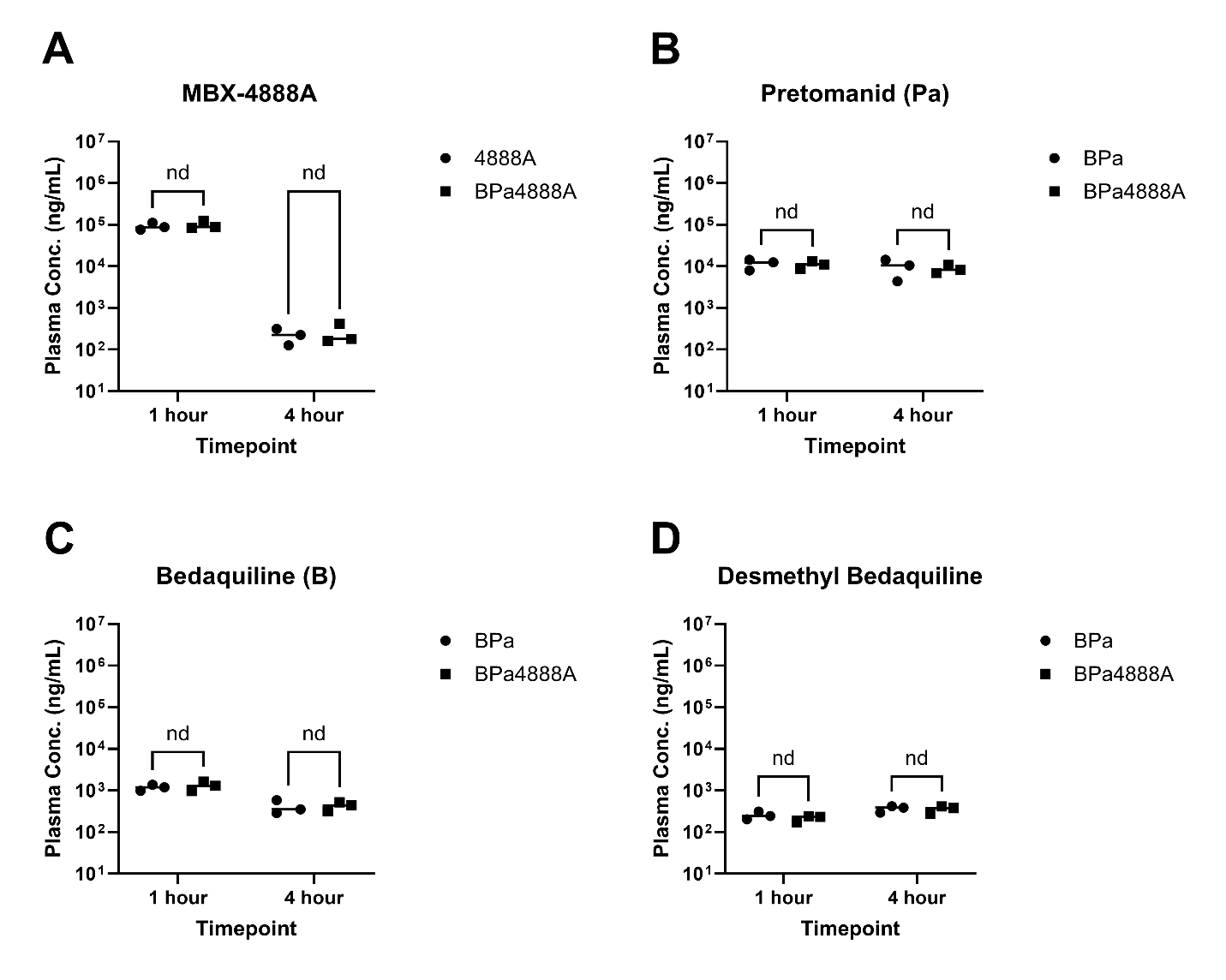


**Figure S2**. Drug exposures in plasma for MBX-4888A (4888A) in combination with bedaquiline (B) and pretomanid (Pa). Plasma was collected from healthy BALB/c mice following a single administration of 4888A monotherapy or combination therapies (BPa and BPa4888A) at 1- and 4-hours post 4888A administration. Drug doses in mg/kg: B (25), Pa (100), 4888A (200). Plasma drug concentrations of MBX-4888A (**A**), pretomanid (**B**), bedaquiline (**C**), and the M2 metabolite of bedaquiline, desmethyl bedaquiline (**D**) were found using LC-MS/MS. Multiple unpaired t test with false discovery rate (Two-stage step-up method of Benjamini, Krieger, and Yekutieli) GraphPad Prism 10.4.1. * Indicates a discovery (q < Q), and “nd” indicates not a discovery (q ≥ Q).

| **Table S2a. Mean plasma concentrations of MBX-4888A following single regimen dosing** | | | |
| --- | --- | --- | --- |
|  | | **Regimen** | |
|  |  | **BPa4888A** | **4888A** |
| **Timepoint** | **1hr** | 99,200 ± 22,472 | 91,667 ± 18,478 |
|  | **4hr** | 253 ± 142 | 222 ± 93 |
| Found plasma concentration of 4888A in ng/mL (± SD) from healthy BALB/c mice. | | | |
| Drug doses in mg/kg: B (25), Pa (100), 4888A (200). | | | |
| Abbreviations: 4888A = MBX-4888A, B = bedaquiline, Pa = pretomanid | | | |
|  |  |  |  |
|  |  |  |  |
| **Table S2b. Mean plasma concentrations of pretomanid following single regimen dosing** | | | |
|  | | **Regimen** | |
|  |  | **BPa4888A** | **BPa** |
| **Timepoint** | **1hr** | 11,020 ± 2176 | 11,620 ± 3309 |
|  | **4hr** | 8,673 ± 1967 | 9,767 ± 5040 |
| Found plasma concentration of pretomanid in ng/mL (±SD) from healthy BALB/c mice. | | | |
| Drug doses in mg/kg: B (25), Pa (100), 4888A (200). | | | |
| Abbreviations: 4888A = MBX-4888A, B = bedaquiline, Pa = pretomanid | | | |
|  |  |  |  |
|  |  |  |  |
| **Table S2c. Mean plasma concentrations of bedaquiline following single regimen dosing** | | | |
|  | | **Regimen** | |
|  |  | **BPa4888A** | **BPa** |
| **Timepoint** | **1hr** | 1,299 ± 336 | 1,182 ± 193 |
|  | **4hr** | 424 ± 105 | 408 ± 157 |
| Found plasma concentration of bedaquiline in ng/mL (±SD) from healthy BALB/c mice. | | | |
| Drug doses in mg/kg: B (25), Pa (100), 4888A (200). | | | |
| Abbreviations: 4888A = MBX-4888A, B = bedaquiline, Pa = pretomanid | | | |
|  |  |  |  |
|  |  |  |  |
| **Table S2d. Mean plasma concentrations of desmethyl bedaquiline* following single regimen dosing** | | | |
|  | | **Regimen** | |
|  |  | **BPa4888A** | **BPa** |
| **Timepoint** | **1hr** | 215 ± 39 | 354 ± 75 |
|  | **4hr** | 253 ± 55 | 367 ± 66 |
| Found plasma concentration of desmethyl Bedaquiline in ng/mL (±SD) from healthy BALB/c mice. | | | |
| Drug doses in mg/kg: B (25), Pa (100), 4888A (200). | | | |
| Abbreviations: 4888A = MBX-4888A, B = bedaquiline, Pa = pretomanid | | | |
| *desmethyl bedaquiline is the M2 metabolite of bedaquiline | | | |

**Methods**

**Murine Infection Models.** *M. tuberculosis* (*Mtb*) strain Erdman (TMCC 107) was used in these infection studies. The Colorado State University Institutional Animal Care and Use Committee (IACUC) approved all procedures used herein. Mice were housed in an ABSL3 facility and were provided *ad libitum* access to food and water. Aerosol infection was performed using a Glas-Col inhalation exposure system using previously calibrated frozen stocks of *Mtb* Erdman. Eight- to ten-week-old mice were obtained from Jackson Laboratories (Bar Harbor, ME). One day after the high-dose aerosol infection a subset of mice were sacrificed to determine the initial bacterial burden in the lungs and was found to be approximately 4.04 log10 CFU (~10,975 CFU). Mice exhibiting clinical symptoms of illness were humanely euthanized.

**Antimicrobial preparation and administration to mice.** Bedaquiline fumarate salt (B) was obtained from Pharmablock (to account for the fumarate salt a 1.2 conversion factor was employed) and was prepared in 20% 2-hydroxylpropy-b-cyclodextrin (Sigma) with acidification to pH 2.5 - 3.0 using 1N HCL; after overnight on a stir plate the pH was measured and adjusted to 3.5. Pretomanid (Pa) was obtained from ChemShuttle and was prepared in 2-hydroxypropyl-β-cyclodextrin (Sigma) with overnight shaking on a rotary shaker, then sonicated in an ice bath for 10 minutes (50% duty cycle (pulses) at 25% amplitude). Finally, 10% w/v of frozen soybean lecithin (MP Bio) was added, stirred for 10 min at room temperature and then sonicated for 15 min in an ice bath (30% amplitude). Linezolid (L) was obtained from LKT Technology Labs and prepared by first incorporating into PEG-200 (Sigma) using a mortar and pestle and then transferred to a conical tube where 0.5% methyl cellulose (Sigma) was added so that the final ratio of PEG-200 to methyl cellulose was 5:95 v/v. MBX-4888A was obtained from Microbiotix and was prepared in a 1:1 equal ratio of Normosol-R to sterile water. Drugs were prepared to their respective concentration needed to achieve a final dose of 25 mg/kg (B), 100 mg/kg (Pa), 100 mg/kg (L), and 200 mg/kg (4888A). Drugs were prepared in weekly batches and kept at 4℃ and aliquoted for daily use. Drugs were administered 5 days per week (Monday through Friday) by oral gavage once daily, given individually in a 200 µl volume with a minimum of 1 hour separating the doses, with the exception of 4888A which was given by subcutaneous injection in a 200 µl volume.

**Drug efficacy experiments and bacterial enumeration.** Drug efficacy determinations were based on lung CFU counts from whole lungs aseptically harvested 5-days after the last day of dosing to allow drug clearance from tissues, or 3 months following the last day of dosing for the relapse arm of the study. Tissues were homogenized (Precellys, Bertin Instruments, Rockville, MD) in PBS with 10% bovine serum albumin to help prevent drug carryover and serially diluted. Portions of these homogenates were plated for CFU on 7H11-OADC agar (i.e., Middlebrook 7H11 agar plates supplemented 0.2% [v:v] glycerol, 10% [v:v] oleic acid-albumin-dextrose-catalase (OADC) supplement, and 0.01 mg/mL cycloheximide, and 0.05 mg/mL carbenicillin). Colonies were enumerated after at least 28 days of incubation at 37°C and plates were incubated for ≥ 6 weeks to ensure all viable colonies were detected. Mice were euthanized by CO_2_ inhalation followed by cervical dislocation, a method approved by the IACUC at Colorado State University.

**Statistical analysis***.* The viable CFU counts per organ were log transformed and evaluated by a one-way analysis of variance (ANOVA) with multiple comparison using either Tukey’s test (pairwise comparison between all treatment groups) or Dunnett’s test (for comparison of each treatment to the start of treatment controls) using Prism 10 (GraphPad Software, San Diego, CA). Differences in relapse proportions were assessed by Fisher’s Exact test using the Holm-Bonferroni correction for multiple comparisons. Differences were considered significant at the 95% level of confidence.

**Pharmacokinetics of MBX-4888A.** B (NIH/HIV reagent Program*), Pa (ChemShuttle), and 4888A (St. Jude Children’s Research Hospital) were prepared as described above. Individual 4888A monotherapy and the combinations (BPa and BPa4888A) were dosed to 3 healthy BALB/c mice for each group. B, and Pa formulations were administered individually by oral gavage in a 0.2 mL volume, while 4888A was administered by subQ injection. B was dosed first followed one-hour later by Pa, and then one-hour later with 4888A. Blood was collected 1- and 4-hours post 4888A administration in Sarstedt Microvette 200 EDTA K3E tubes. Plasma was separated through centrifugation at 3,000 rcf for 5 minutes at 4 ℃. Plasma was stored at -80℃ or on dry ice prior to LC-MS/MS analysis.

***Bedaquiline source acknowledgment.** The following reagent was obtained through the NIH HIV Reagent Program, Division of AIDS, NIAID, NIH: Bedaquiline Fumarate, ARP-12702, contributed by Janssen Pharmaceuticals.

**LC-MS/MS Analytical Methods.** MBX-4888 was analyzed by LC-MS/MS according to previously published methods (1). For Bedaquiline (HIV Reagent Program), Des-methyl Bedaquiline (Clearsynth), or Pretomanid (Bioduro) neat 1mg/mL DMSO stocks of were serial diluted in 50/50 Acetonitrile (ACN)/ Milli-Q water to create standard curve spiking solutions. Drug free CD-1 mouse standards were created by adding 10µLs of spiking solutions to 90µLs of drug free plasma (CD-1 K2EDTA Mouse, Bioreclamation IVT). 10µLs of control, standard, or study sample were added to 100 µLs of ACN protein precipitation solvent containing 10 ng/mL of the internal standards BDQ-d6 (Toronto Research Chemical) and PMD-d4 (Toronto Research Chemical). Extracts were vortexed for 5 minutes and centrifuged at 4000 RPM for 5 minutes. The extract supernatant was transferred for HPLC-MS/MS analysis. LC-MS/MS analysis was performed on a Sciex Applied Biosystems Qtrap 6500+ triple-quadrupole mass spectrometer coupled to a Shimadzu Nexera X2 UHPLC system to quantify each drug in plasma. Chromatography was performed on an Agilent SB-C8 (2.1x30 mm; particle size, 3.5µm) using a reverse phase gradient. Milli-Q deionized water with 0.1% formic acid was used for the aqueous mobile phase and 0.1% formic acid in ACN for the organic mobile phase. Multiple-reaction monitoring of parent/daughter transitions in electrospray positive-ionization mode was used to quantify all the analytes. The following MRM transitions were used for detection of BDQ (555.00/58.00), BDQ-M2 (541.00/480.00), PMD (360.00/175.00), PMD-d4 (364.00/175.00), and BDQ-d6 (561.00/64.00). Sample analysis was accepted if the concentrations of the quality control samples were within 20% of the nominal concentration. Data processing was performed using Analyst software (version 1.6.2; Applied Biosystems Sciex).

**References**

1. Bauman AA, Sarathy JP, Kaya F, Massoudi LM, Scherman MS, Hastings C, Liu J, Xie M, Brooks EJ, Ramey ME, Jones IL, Benedict ND, Maclaughlin MR, Miller-Dawson JA, Waidyarachchi SL, Butler MM, Bowlin TL, Zimmerman MD, Lenaerts AJ, Meibohm B, Gonzalez-Juarrero M, Lyons MA, Dartois V, Lee RE, Robertson GT. 2024. Spectinamide MBX-4888A exhibits favorable lesion and tissue distribution and promotes treatment shortening in advanced murine models of tuberculosis. Antimicrob Agents Chemother 68:e0071624.
